# Bacterial Profile and Antibiotic Susceptibility Patterns of Urogenital Isolates Among Women With Preterm Labor at a Tertiary Hospital in Southwestern Uganda

**DOI:** 10.7759/cureus.114241

**Published:** 2026-08-09

**Authors:** Wilson Birungi, Stuart Turanzomwe, Onesmus Byamukama, Stephen Bawakanya, Francis Orishaba, Taseera Kabanda, Musa Kayondo, Joseph Ngonzi, Rogers Kajabwangu

**Affiliations:** 1 Obstetrics and Gynecology, Mbarara University of Science and Technology, Mbarara, UGA; 2 Obstetrics and Gynecology, Kabale University School of Medicine, Kabale, UGA; 3 Microbiology and Parasitology, Mbarara University of Science and Technology, Mbarara, UGA; 4 Obstetrics and Gynecology, Mbarara Regional Referral Hospital, Mbarara, UGA

**Keywords:** antibiotic susceptibility, bacterial isolates, bacterial profile, genital tract, preterm labor, south-western uganda, urinary tract, urogenital bacterial colonization

## Abstract

Background: Pathogenic bacterial colonization of the urogenital tract during pregnancy may lead to infection and inflammation, contributing to adverse obstetric and neonatal outcomes such as preterm labor, preterm premature rupture of membranes, chorioamnionitis, neonatal sepsis, and perinatal mortality. Early identification of bacterial isolates and their antibiotic susceptibility patterns is essential for guiding appropriate antimicrobial therapy, reducing maternal and neonatal morbidity, and informing local treatment guidelines. This study aimed to determine the bacterial isolates colonizing the urogenital tract and their antibiotic susceptibility patterns among women presenting with preterm labor at Mbarara Regional Referral Hospital.

Methods: This cross-sectional study consecutively enrolled 156 women with preterm labor between November 2022 and April 2023. Midstream urine samples, endocervical swabs, and high vaginal swabs were collected for bacterial culture, identification, and antibiotic susceptibility testing. Data were analyzed descriptively, and bacterial isolates and antibiotic susceptibility patterns were summarized as frequencies and percentages.

Results: Bacteria were isolated from urine samples in 43/156 (27.6%) participants and from genital tract specimens in 75/156 (48.1%). The predominant urinary isolates were *Klebsiella* spp. (20/43, 46.5%) and *Staphylococcus aureus* (16/43, 37.2%), while *Escherichia coli* accounted for 4/43 (9.3%) of urinary isolates. In the genital tract, *S. aureus* was the most frequently isolated organism (43/75, 57.3%), whereas *Proteus* spp. (1/75, 1.3%) and *Pseudomonas* spp. (1/75, 1.3%) were the least common. The predominant urinary and genital tract isolates demonstrated high resistance to ampicillin and azithromycin but were generally susceptible to ceftriaxone, gentamicin, and ciprofloxacin.

Conclusion: *Staphylococcus aureus* and *Klebsiella* spp. were the predominant urogenital bacterial isolates among women with preterm labor. Most bacterial isolates were susceptible to gentamicin, ciprofloxacin, ceftriaxone, and ceftazidime but demonstrated high resistance to ampicillin and azithromycin. These findings highlight the need for routine culture and antibiotic susceptibility testing and may inform future updates of empirical antibiotic treatment guidelines in Uganda.

## Introduction

Pregnancy is associated with physiological, hormonal, and immunological adaptations that favor bacterial colonization of the urinary and genital tracts [1]. When pathogenic bacteria are present, they may ascend the genital tract and stimulate inflammatory pathways that contribute to preterm labor with births occurring before 37 weeks of gestation [2]. The physiological, hormonal, and immunological adaptations that favor bacterial colonization of the urinary and genital tracts may result in preterm labor [3]. Other adverse pregnancy outcomes include chorioamnionitis, neonatal sepsis, and perinatal mortality [4,5].

The bacterial profile among women with preterm labor is polymicrobial and varies across geographical regions. Commonly reported urogenital isolates include *Staphylococcus aureus*, *Klebsiella pneumoniae*, *Escherichia coli*, *Enterococcus faecalis*, *Pseudomonas aeruginosa*, and *Proteus* species [6,7]. Because the distribution of bacterial isolates differs between populations, knowledge of the local bacterial profile is essential for understanding the microorganisms commonly associated with preterm labor in a given setting.

In addition, increasing antimicrobial resistance among urogenital bacterial isolates poses a major challenge to the empirical use of antibiotics during pregnancy [7,8]. Antibiotic susceptibility testing provides important information for selecting effective antimicrobial agents, promotes rational antibiotic use, and supports antimicrobial stewardship. Local susceptibility data are particularly important because resistance patterns vary geographically and change over time.

Despite the clinical importance of these data, information on the bacterial profile and antibiotic susceptibility patterns of urogenital isolates among women with preterm labor in Uganda remains scarce. This study therefore aimed to identify bacterial isolates from the urinary and genital tracts and determine their antibiotic susceptibility patterns among women presenting with preterm labor at Mbarara Regional Referral Hospital, Uganda.

## Materials and methods

Study stetting, design, and population

This was a cross-sectional study conducted among women with preterm labor at the maternity ward of Mbarara Regional Referral Hospital (MRRH). MRRH is a tertiary hospital owned and funded by the government of Uganda through the Ministry of Health. It provides care to about 12,000 pregnant women annually, including those with preterm labor [9]. Our study population comprised women admitted with preterm labor to the maternity ward of MRRH. We included all women with a diagnosis of preterm labor evidenced by the presence of two or more palpable uterine contractions in 10 minutes and cervical dilatation ≥2 cm at a gestational age of 26-36 weeks and six days that was calculated from the first day of the last normal menstrual period or first trimester ultrasound scan.

Sample size and sampling

The sample size was calculated using OpenEpi online software [10] for estimating a single population proportion. A finite population correction was applied based on an estimated population of 240 women with preterm labor expected to present to Mbarara Regional Referral Hospital over a six-month period. The expected prevalence of bacterial colonization was assumed to be 67.8% based on a study conducted among women with preterm labor in India [11]. After accounting for a 10% non-response rate, the final sample size was 156 participants. Eligible women were enrolled consecutively until the required sample size was achieved.

Sample collection and processing

Informed consent was obtained from each woman before a sample was collected. The women were taken to a private clinical room one at a time and instructed on how to collect clean-catch midstream urine samples. Then, a high vaginal swab and endocervical swab were subsequently collected aseptically by the principal investigator or trained research assistants. Samples were transported to the laboratory within 30 minutes of collection for microbiological culture and bacterial identification. For quality assurance, duplicate specimens from every 10th participant were analyzed at an independent reference laboratory. Laboratory results were communicated to the clinical team to facilitate appropriate management of participants with positive bacterial cultures.

Identification and biochemical characterization of bacteria

Bacterial isolation and identification were performed using conventional culture, phenotypic, and biochemical methods. Urine, high vaginal, and endocervical specimens were inoculated onto appropriate culture media, including 5% sheep blood agar, MacConkey agar, mannitol salt agar, and modified Thayer-Martin agar, according to standard microbiological procedures and the organisms under investigation. Culture plates were incubated at 37°C for 24-72 hours under appropriate atmospheric conditions, including aerobic incubation and carbon dioxide-enriched conditions for fastidious organisms, where indicated. Bacterial isolates were identified using Gram staining and standard biochemical tests. Gram-positive cocci were characterized using catalase, coagulase, and DNase tests, while Gram-negative bacilli were identified using citrate utilization, urease, oxidase, and triple sugar iron (TSI) tests, as appropriate.

Antibiotic susceptibility

Antimicrobial susceptibility testing was performed using the Kirby-Bauer disk diffusion method on Mueller-Hinton agar for non-fastidious organisms and chocolate agar for fastidious organisms following standard laboratory procedures routinely employed at the microbiology laboratory of Mbarara Regional Referral Hospital. A standardized 0.5 McFarland bacterial suspension was inoculated onto the appropriate culture medium, and antibiotic discs were selected according to the bacterial species. The antibiotic panel included ampicillin, azithromycin, ceftriaxone, ciprofloxacin, gentamicin, and ceftazidime because these agents are commonly used in obstetric and gynecological practice in Uganda, including the treatment of women with preterm prelabor rupture of membranes [12]. Following incubation at 37°C for 24-48 hours under appropriate atmospheric conditions, inhibition zone diameters were measured and interpreted as susceptible, intermediate, or resistant according to the Clinical and Laboratory Standards Institute (CLSI) Performance Standards for Antimicrobial Susceptibility Testing (M100) [13]. Internal quality control was performed using *Staphylococcus aureus* ATCC 25923 and *Escherichia coli* ATCC 25922 reference strains.

Statistical analysis

Data were analyzed using descriptive statistics. All tests were analyzed using STATA version 17.0 (StataCorp LLC, College Station, TX, USA) [14]. The frequencies of bacterial isolates from the urogenital tract of women with preterm labor were obtained and presented in tables. The susceptibility patterns of the cultured bacteria to the tested antibiotics were expressed as frequencies and percentages for a given bacterial isolate.

## Results

A total of 156 women with preterm labor were enrolled. The mean age was 25.5 ± 5.8 years, and most participants were aged 20-34 years (126, 80.8%). The majority had parity <4 (137, 87.8%), were referred from other health facilities (120, 76.9%), were HIV-negative (144, 92.3%), and reported abnormal vaginal discharge (117, 75.0%). Most women presented with late preterm labor (98, 62.8%) and had undergone two or fewer vaginal examinations (115, 73.7%). The baseline sociodemographic, obstetric, and clinical characteristics of the participants are presented in Table 1.

**Table 1:** Baseline sociodemographic, obstetric, and clinical characteristics of women presenting with preterm labor at Mbarara Regional Referral Hospital (N = 156). ANC: antenatal care; HIV: human immunodeficiency virus; SD: standard deviation.

Characteristics	n (%)
Age, years (mean ± SD)	25.5 ± 5.8
<20	17 (10.9)
20-34	126 (80.8)
≥35	13 (8.3)
Parity	
<4	137 (87.8)
≥4	19 (12.2)
Residence	
Urban area	65 (41.7)
Rural area	91 (58.3)
Highest level of education	
Primary	77 (49.4)
Secondary	54 (34.6)
Tertiary/university	25 (16.0)
Multiple sexual partners	
No	148 (94.9)
Yes	8 (5.1)
Referral status	
No	36 (23.1)
Yes	120 (76.9)
HIV status	
Negative	144 (92.3)
Positive	12 (7.7)
Diabetes mellitus	
No	153 (98.1)
Yes	3 (1.9)
Antibiotic use in the preceding 7 days	
No	128 (82.1)
Yes	28 (17.9)
Abnormal vaginal discharge	
No	39 (25.0)
Yes	117 (75.0)
Gestational age	
Early preterm (<34 weeks)	58 (37.2)
Late preterm (34-36 weeks)	98 (62.8)
Number of ANC visits	
<4	78 (50.0)
≥4	78 (50.0)
Premature rupture of membranes	
No	83 (53.2)
Yes	73 (46.8)
Number of vaginal examinations	
≤2	115 (73.7)
>2	41 (26.3)

Urinary tract bacterial profile

Bacteria were isolated from the urine samples of 43 (27.6%) of the 156 women with preterm labor. *Klebsiella* spp. was the predominant urinary isolate, accounting for 20 (46.5%) of all urinary isolates, followed by *S. aureus* with 16 (37.2%) isolates. *Escherichia coli* was identified in 4 (9.3%) participants, while *Proteus* spp., *Pseudomonas* spp., and *Enterobacter* spp. were each isolated from 1 (2.3%) participant (Table 2).

**Table 2:** Distribution of bacterial isolates recovered from the urine samples of women with preterm labor (N=43).

Bacterial isolate	Number of isolates	Percentage
*Klebsiella* spp.	20	46.5
Staphylococcus aureus	16	37.2
Escherichia coli	4	9.3
*Proteus* spp.	1	2.3
*Enterobacter* spp.	1	2.3
*Pseudomonas* spp.	1	2.3
Total	43	100.0

Genital tract bacterial profile

A total of 75 bacterial isolates were recovered from the genital tract specimens of women with preterm labor. *Staphylococcus aureus* was the predominant isolate, accounting for 43 (57.3%) of all genital tract isolates, followed by *Klebsiella* spp. with 18 (24.0%) isolates and *Enterococcus* spp. with 8 (10.7%) isolates. *Streptococcus viridans* and *E. coli* each accounted for 2 (2.7%) isolates, while *Proteus* spp. and *Pseudomonas* spp. were the least frequently isolated organisms, each contributing 1 (1.3%) isolate (Table 3).

**Table 3:** Distribution of bacterial isolates recovered from the genital tract specimens of women with preterm labor (N=75).

Bacterial isolate	Number of isolates	Percentage
Staphylococcus aureus	43	57.3
*Klebsiella* spp.	18	24.0
*Enterococcus* spp.	8	10.7
Streptococcus viridans	2	2.7
Escherichia coli	2	2.7
*Proteus* spp.	1	1.3
*Pseudomonas* spp.	1	1.3
Total	75	100.0

Antibiotic susceptibility patterns of urinary bacterial isolates

The antimicrobial susceptibility patterns of urinary bacterial isolates are presented in Table 4. Among the predominant urinary isolates, *Klebsiella* spp. demonstrated the highest resistance to ampicillin (11, 55.0%) and azithromycin (10, 50.0%), while showing the greatest susceptibility to gentamicin (14, 70.0%), ciprofloxacin (12, 60.0%), and ceftazidime (12, 60.0%). *Staphylococcus aureus* was completely resistant to ampicillin (16, 100.0%) but remained highly susceptible to ciprofloxacin and gentamicin (13, 81.3% each), with moderate susceptibility to ceftriaxone (10, 62.5%) and ceftazidime (8, 50.0%).

**Table 4:** Antimicrobial susceptibility patterns of urinary bacterial isolates among women with preterm labor. S: susceptible; I: intermediate; R: resistant.

Organism antibiotic	Susceptibility pattern	*Klebsiella* (n=20)	*Staphylococcus aureus *(n=16)	*Proteus* spp. (n=1)	*Escherichia coli *(n=4)	*Pseudomonas* spp. (n=1)	*Enterobacter *spp. (n=1)
Ceftriaxone, n (%)	S	11 (55.0)	10 (62.5)	0 (0.0)	3 (75.0)	0 (0.0)	0 (0.0)
	I	8 (40.0)	3 (18.8)	0 (0.0)	0 (0.0)	0 (0.0)	0 (0.0)
	R	1 (5.0)	3 (18.8)	1 (100.0)	1 (25)	1 (100.0)	1 (100.0)
Ciprofloxacin, n (%)	S	12 (60.0)	13 (81.3)	1 (100.0)	4 (100.0)	0 (0.0)	0 (0.0)
	I	7 (35.0)	0 (0.0)	0 (0.0)	0 (0.0)	1 (100.0)	0 (0.0)
	R	1 (5.0)	3 (18.8)	0 (0.0)	0 (0.0)	0 (0.0)	1 (100.0)
Ampicillin, n (%)	S	4 (20.0)	0 (0.0)	0 (0.0)	1 (25.0)	0 (0.0)	0 (0.0)
	I	5 (25.0)	0 (0.0)	0 (0.0)	0 (0.0)	0 (0.0)	1 (100.0)
	R	11 (55.0)	16 (100.0)	1 (100.0)	3 (75.0)	1 (100.0)	0 (0.0)
Azithromycin, n (%)	S	8 (40.0)	8 (50.0)	0 (0.0)	0 (0.0)	0 (0.0)	0 (0.0)
	I	2 (10.0)	0 (0.0)	0 (0.0)	0 (0.0)	0 (0.0)	0 (0.0)
	R	10 (50.0)	8 (50.0)	1 (100.0)	4 (100.0)	1 (100.0)	1 (100.0)
Gentamicin, n (%)	S	14 (70.0)	13 (81.3)	1 (100.0)	4 (100.0)	0 (0.0)	1 (100.0)
	I	2 (10.0)	1 (6.3)	0 (0.0)	0 (0.0)	0 (0.0)	0 (0.0)
	R	4 (20.0)	2 (12.5)	0 (0.0)	0 (0.0)	1 (100.0)	0 (0.0)
Ceftazidime, n (%)	S	12 (60.0)	8 (50.0)	1 (100.0)	4 (100.0)	0 (0.0)	1 (100.0)
	I	5 (25.0)	3 (18.8)	0 (0.0)	0 (0.0)	1 (100.0)	0 (0.0)
	R	3 (15.0)	5 (31.3)	0 (0.0)	0 (0.0)	0 (0.0)	0 (0.0)

Susceptibility in the genital tract

The antimicrobial susceptibility patterns of genital tract bacterial isolates are presented in Table 5. *Klebsiella* spp. demonstrated the highest resistance to ampicillin (14, 77.8%) and azithromycin (10, 55.6%) while remaining highly susceptible to ceftriaxone (16, 88.9%), ciprofloxacin (16, 88.9%), and gentamicin (15, 83.3%). *Staphylococcus aureus* was completely resistant to ampicillin (43, 100.0%) and also demonstrated substantial resistance to ceftazidime (26, 60.5%), whereas high susceptibility was observed to ciprofloxacin (37, 86.0%), gentamicin (36, 83.7%), and ceftriaxone (33, 76.7%). Susceptibility patterns for the remaining bacterial species should be interpreted cautiously because only a few isolates were recovered.

**Table 5:** Antimicrobial susceptibility patterns of genital tract bacterial isolates among women with preterm labor. S: susceptible; I: intermediate; R: resistant.

Organism antibiotic	Susceptibility pattern	*Klebsiella* (n=18)	*Staphylococcus aureus* (n=43)	*Streptococcus viridans* (n=2)	*Enterococcus* spp. (n=8)	*Proteus* spp. (n=1)	*Escherichia coli* (n=2)	*Pseudomonas *spp. (n=1)
Ceftriaxone, n (%)	S	16 (88.9)	33 (76.7)	1 (50.0)	7 (87.5)	1 (100.0)	2 (100.0)	0 (0.0)
	I	1 (5.6)	6 (14.0)	0 (0.0)	0 (0.0)	0 (0.0)	0 (0.0)	1 (100.0)
	R	1 (5.6)	4 (9.3)	1 (50.0)	1 (12.5)	0 (0.0)	0 (0.0)	0 (0.0)
Ciprofloxacin, n (%)	S	16 (88.9)	37 (86.0)	2 (100.0)	5 (62.8)	1 (100.0)	2 (100.0)	1 (100.0)
	I	1 (5.6)	1 (2.3)	0 (0.0)	0 (0.0)	0 (0.0)	0 (0.0)	0 (0.0)
	R	1 (5.6)	5 (11.6)	0 (0.0)	3 (37.5)	0 (0.0)	0 (0.0)	0 (0.0)
Ampicillin, n (%)	S	3 (16.7)	0 (0.0.0)	0 (0.0)	0 (0.0)	0 (0.0)	1 (50.0)	0 (0.0)
	I	1 (5.6)	0 (0.0)	2 (100.0)	7 (88.0)	0 (0.0)	0 (0.0)	0 (0.0)
	R	14 (77.8)	43 (100.0)	0 (0.0)	1 (12.0)	1 (100.0)	1 (50.0)	1 (100.0)
Azithromycin, n (%)	S	4 (22.2)	31 (72.1)	1 (50.0)	5 (63.0)	0 (0.0)	0 (0.0)	0 (0.0)
	I	4 (22.2)	0 (0.0)	0 (0.0)	0 (0.0)	0 (0.0)	0 (0.0)	0 (0.0)
	R	10 (55.6)	12 (27.9)	1 (50.0)	3 (37.0)	1 (100.0)	2 (100.0)	1 (100.0)
Gentamicin, n (%)	S	15 (83.3)	36 (83.7)	1 (50.0)	5 (63.0)	1 (100.0)	2 (100.0)	0 (0.0)
	I	2 (11.1)	4 (9.3)	0 (0.0)	0 (0.0)	0 (0.0)	0 (0.0)	1 (100.0)
	R	1 (5.6)	3 (7.0)	1 (50.0)	3 (37.0)	0 (0.0)	0 (0.0)	0 (0.0)
Ceftazidime, n (%)	S	13 (72.2)	16 (37.2)	1 (50.0)	5 (63.0)	1 (100.0)	2 (100.0)	0 (0.0)
	I	3 (16.7)	1 (2.3)	0 (0.0)	1 (12.0)	0 (0.0)	0 (0.0)	0 (0.0)
	R	2 (11.1)	26 (60.5)	1 (50.0)	2 (25.0)	0 (0.0)	0 (0.0)	1 (100.0)

## Discussion

This study described the bacterial profile and antimicrobial susceptibility patterns of urogenital bacterial isolates among women presenting with preterm labor at a tertiary hospital in southwestern Uganda. *Staphylococcus aureus* and *Klebsiella* spp. were the predominant organisms isolated from the genital and urinary tracts, respectively. The majority of isolates demonstrated high in vitro susceptibility to ceftriaxone, gentamicin, and ciprofloxacin but exhibited substantial resistance to ampicillin and azithromycin.

The predominant bacteria isolated from the urogenital tract were *S. aureus* (50.0%) and *Klebsiella* spp. (30.2%), while *Enterococcus* spp. (7.5%), *E. coli* (4.7%), *Proteus* spp. (1.9%), *Pseudomonas* spp. (1.9%), and *Enterobacter* spp. (0.9%) were isolated less frequently. *Klebsiella* spp. were the predominant urinary isolates, whereas *S. aureus* predominated in genital tract specimens. These findings are consistent with studies conducted at the Mulago National Referral Hospital and Mbarara Regional Referral Hospital, which similarly identified *Klebsiella* spp. and *S. aureus* among the predominant organisms isolated from urine and genital tract specimens of pregnant women [7,15]. Likewise, Tumuhamye et al. [16] reported these organisms among the common urogenital bacterial isolates in a multisite study conducted in central Uganda. The similarity in findings may reflect comparable study populations, healthcare settings, and regional bacterial ecology, as all studies were conducted in Ugandan tertiary referral hospitals.

The predominance of *S. aureus* in genital tract specimens is also comparable to findings reported among women in labor in India [11]. Although *S. aureus* commonly colonizes the skin and mucosal surfaces, it possesses several virulence factors, including microbial surface components recognizing adhesive matrix molecules (MSCRAMMs), which facilitate adherence to epithelial tissues and persistence within the urogenital tract [17]. Similarly, *Klebsiella* spp. possesses a polysaccharide capsule, fimbrial adhesins, and other virulence determinants that enhance colonization, evade host immune responses, and promote persistence within the urinary tract [18,19]. In addition, the predominance of *Klebsiella* spp. in urine cultures may be explained by the close anatomical proximity of the urinary and gastrointestinal tracts, which facilitates ascending colonization by enteric Gram-negative organisms. Pregnancy-related physiological changes, including urinary stasis, ureteric dilatation, and altered immune responses, further increase susceptibility to bacterial colonization [20,21].

Contrary to our findings, studies conducted in India, Rwanda, Kenya, and Ethiopia identified *E. coli* as the predominant urogenital isolate among pregnant women [6,22-24]. These differences may be attributed to variations in study populations, geographical settings, laboratory methods, and participant characteristics. Unlike this study, which exclusively enrolled women with preterm labor, the Rwandan study included predominantly women without preterm labor, the Kenyan study focused on women living with HIV, and the Ethiopian study enrolled women with gestational ages of at least 35 weeks, including women at term. Such differences are likely to influence the spectrum of bacterial colonization observed.

The predominant urogenital bacterial isolates demonstrated high levels of resistance to ampicillin and azithromycin, whereas susceptibility to ceftriaxone, gentamicin, and ciprofloxacin remained relatively high. Among urinary isolates, *Klebsiella* spp. exhibited the highest resistance to azithromycin (63.2%) and ampicillin (57.9%), while *E. coli* demonstrated resistance to ampicillin (75.0%) and azithromycin (100%). Similarly, *S. aureus* demonstrated high susceptibility to ciprofloxacin and gentamicin (81.3% each), with moderate susceptibility to ceftriaxone (62.5%), ceftazidime (50.0%), and azithromycin (50.0%). These findings are comparable to those reported among pregnant women with culture-positive urinary tract infections at the Mbarara Regional Referral Hospital and in Ethiopia, where high resistance to ampicillin and other commonly prescribed antibiotics was observed [7,25].

Similarly, genital tract isolates demonstrated high resistance to ampicillin and azithromycin. *Klebsiella* spp. showed resistance rates of 76.9% and 53.8% to ampicillin and azithromycin, respectively, whereas *S. aureus* exhibited substantial resistance to ceftazidime (53.5%) and azithromycin (32.6%). Nevertheless, *Klebsiella* spp. retained high susceptibility to ceftriaxone (84.6%), ciprofloxacin (84.6%), and ceftazidime (84.6%). These findings are generally consistent with those reported by Tumuhamye et al. [16], who also observed good activity of ceftriaxone, gentamicin, and ciprofloxacin against common urogenital pathogens isolated from women in labor.

The high resistance observed to ampicillin and azithromycin is likely attributable to the long-standing and widespread use of these antibiotics for empirical treatment of bacterial infections, resulting in sustained antimicrobial selective pressure. In addition, inappropriate antibiotic prescribing, self-medication, incomplete treatment courses, and unrestricted access to antibiotics without a prescription may further accelerate the emergence and dissemination of resistant bacterial strains [26]. Conversely, the relatively preserved susceptibility to ceftriaxone, gentamicin, and ciprofloxacin suggests that these antibiotics continue to retain in vitro activity against the predominant urogenital pathogens in our setting, although their use during pregnancy should remain guided by antimicrobial susceptibility testing, obstetric safety considerations, and antimicrobial stewardship principles.

Overall, these findings underscore the importance of routine microbiological culture and antimicrobial susceptibility testing, where feasible, to guide appropriate antibiotic selection among women presenting with preterm labor. In our setting, continuous surveillance of bacterial profiles and antimicrobial resistance patterns is essential to inform empirical treatment guidelines, support antimicrobial stewardship programs, and mitigate the growing threat of antimicrobial resistance in maternal healthcare settings.

Strengths

This study provides valuable data on the bacterial profile and antimicrobial susceptibility patterns of urogenital isolates among women with preterm labor, a population for whom such data are limited in Uganda. Unlike previous studies that focused on either urinary or genital tract isolates, this study simultaneously evaluated bacteria recovered from urine, high vaginal, and endocervical specimens, providing a comprehensive assessment of urogenital bacterial colonization. In addition, antimicrobial susceptibility testing was performed using standardized microbiological methods, generating locally relevant evidence that may inform empirical antibiotic therapy and antimicrobial stewardship in the management of women with preterm labor when indicated.

Limitations

This study was conducted at a single tertiary referral hospital and used conventional culture techniques, which may limit the generalizability of the findings and underestimate fastidious and non-culturable microorganisms. However, the standardized microbiological procedures employed and the inclusion of both urinary and genital tract specimens provide robust and locally relevant evidence on the bacterial profile and antimicrobial susceptibility patterns among women with preterm labor in southwestern Uganda. Additionally, only antibiotics routinely used in obstetric and gynecological practice and available for susceptibility testing at the Mbarara Regional Referral Hospital were evaluated. Consequently, susceptibility patterns for other clinically relevant antibiotics were not assessed, which may limit comparisons with studies using broader antimicrobial panels. Finally, residual confounding from unmeasured factors that may influence bacterial colonization and antimicrobial susceptibility cannot be excluded.

## Conclusions

*Staphylococcus aureus* and *Klebsiella* spp. were the predominant urogenital bacterial isolates among women with preterm labor at the Mbarara Regional Referral Hospital. Most isolates demonstrated high in vitro susceptibility to gentamicin, ciprofloxacin, ceftriaxone, and ceftazidime but showed substantial resistance to ampicillin and azithromycin. These findings provide locally relevant evidence that underscores the importance of routine microbiological culture and antimicrobial susceptibility testing to guide antibiotic selection when indicated. However, treatment decisions should be integrated with clinical findings, local antimicrobial stewardship policies, and ongoing antimicrobial surveillance. Larger multicenter studies are warranted to validate these findings and assess their generalizability to other settings.
